# The Short-Term Effect of the COVID-19 Crisis on Employment Probabilities of Labour-Market Entrants in the Netherlands

**DOI:** 10.1007/s10645-022-09406-8

**Published:** 2022-05-04

**Authors:** Henri Bussink, Tobias Vervliet, Bas ter Weel

**Affiliations:** 1 SEO Amsterdam Economics, Amsterdam, The Netherlands; 2 Amsterdam School of Economics, University of Amsterdam, Amsterdam, The Netherlands

**Keywords:** COVID-19 crisis, Employment, Young workers, J10, J23, I24

## Abstract

This research documents employment opportunities of labour-market entrants during the COVID-19 crisis in the Netherlands. Two recent cohorts of graduates are studied and compared to two pre-COVID-19 cohorts: the 2019 cohort was unexpectedly hit by the COVID-19 crisis about six months after entering the labour market and the 2020 cohort graduated and entered the labour market in the midst of a lockdown. Our estimation results suggest short-term effects of lockdowns on employment probabilities, specifically for relatively lower educated labour-market entrants. The effects appear to be relatively small in size and seem to fade when the lockdown measures are eased. Men seem to have suffered more than women and some sectors are hit harder than others, which could result in short-run mismatches. Overall the effects appear to be less severe than during an economic recession, which is most likely due to the tight labour market and the strong measures taken by the government to mitigate the labour-market impact of the COVID-19 crisis.

## Introduction

Graduating and entering the labour market during an economic recession is likely to have a negative effect on employment opportunities in the short run. The COVID-19 crisis is a health crisis that has among others led to economic lockdowns and uncertainty among workers and employers about future employment prospects. Lockdowns and uncertainty are likely to affect labour supply and demand in the short run. Evidence from past recessions suggests that in the short run workers most directly affected are young labour-market entrants who suffer from reductions in employment opportunities.^1^ This could lead to scarring, which is defined as a persistent negative impact on earnings and employment rates of people who enter the labour market during a recession. During the COVID-19 crisis a large package of measures has been implemented to prevent employers from laying off workers, which could mitigate the effects of scarring or shorten the period in which scarring could occur.

This research documents and interprets the employment probabilities of two cohorts of labour-market entrants who entered the labour market just before (2019 cohort) and during (2020 cohort) the COVID-19 crisis. Employment probabilities in the first months after entering the labour market of these two cohorts are compared to the employment probabilities of labour-market entrants in cohorts before the COVID-19 crisis (2017 and 2018 cohorts). The empirical analysis focuses on the labour market in the Netherlands and documents the development of employment probabilities of all labour-market entrants between 16 and 30 years old who left education in the period 2017–2020. For each of the four cohorts labour-market information is available for 170,000 to 180,000 graduates, at different levels of education. We make use of administrative data available through remote access facilities at Statistics Netherlands. We restrict the analysis to labour-market entrants who have finished education and who have received a diploma before the end of October in the year of graduation, and who are available for work. This implies that all graduates from secondary vocational education (MBO), those who graduate from higher vocational education (HBO) and university graduates are included in the analysis. We leave out people who continue studying after having completed some level of education and those who enter the labour market without a diploma or starting qualification (dropouts).

To measure employment probabilities we define employment as having a job as an employee for at least three days a week (part-time work factor of 0.6). If someone has several jobs at the same time, the number of working hours is aggregated to determine whether someone is working at least three days a week. It only concerns jobs that are officially registered, there is no information about informal jobs or self-employed workers within the data at our disposal. Our strategy is to compare and document differences in employment probabilities of labour-market entrants of four cohorts of entrants. To be able to compare the labour-market position of entrants from different cohorts, the employment probabilities are adjusted for differences in composition between cohorts. This concerns differences in the composition by educational level, field of study, gender, migration background and a number of personal and family characteristics.

Our main findings show that during lockdowns the employment probabilities of labour-market entrants drop significantly and that they recover relatively rapidly when the lockdown measures are eased. There is a difference in the development of employment opportunities between higher and lower educated workers during the pandemic, which reveals that higher educated workers do not seem to have suffered whereas lower educated graduates have seen a drop in employment probabilities. This drop seems to be temporary although the labour market is not fully back on trend by June 2021. The 2020 cohort who faced a lockdown upon entrance and who have likely suffered from delays in school or university is different in size and composition from the 2019 cohort, which suggests selective entrance. Especially lower educated graduates from vocational education tracks seem to have (been forced to) delay entrance, perhaps due to the inability to finish a number of educational requirement (such as obligatory internships). Differences between men and women suggest that men have suffered more from the lockdown measures than women in terms of employment opportunities, a pattern which seems to be consistent with employment patterns by sector. Especially in health and governmental services employment opportunities have risen, which are female-dominated sectors.

This research contributes to understanding the short-run effects of graduating during a recession and sheds light on the effects of the measures taken during the COVID-19 crisis on employment probabilities of labour-market entrants. There is a literature on the effects of graduating during a recession, which suggests that there is likely both a short-term and long-term effect on employment opportunities. In a study using US data in the period 1979–1989 Kahn (2010) finds persistent mismatch in the sense that college cohorts who graduate in worse economic times are in lower-level occupations. Oreopoulos et al. (2012) document for Canadian college graduates that graduating in a recession leads to persistent downgrading for college graduates in terms of employment and that less-advantaged college graduates permanently lose access to better employers. Raaum and Røed (2006) find that labour-market conditions in Norway at the time of entry into the labour market have a substantial and persistent effect on adult employment prospects. Andrews et al. (2020) find that graduating in a recession leads to scarring effects on earnings for up to a decade in Australia. Recessions disrupt worker-firm match quality and fade when workers switch to more productive firms. For the Netherlands, Van den Berge (2018) finds that employment effects of graduating in a recession are small in the period 1996–2012. He shows that entry conditions correlate with the quality of the match, which is worse during economic downturns. Job mobility resolves part of the initial mismatch but not for those who graduate at lower levels of vocational education. Our contribution to this literature is empirical in the sense that we document short-term employment effects of a severe fall in economic activity due to economic lockdowns and uncertainty among employers. The size of our findings is small, which are likely to be driven by the measures taken by the government to compensate employers for most of the wage costs. We also find that the effect on employment is temporary which is different from an economic recession where labour demand only recovers gradually. Our findings could be seen as preliminary evidence that the COVID-19 crisis has different labour-market effects compared to economic recessions, at least in the short run.

The research on the short-term effects on employment of the COVID-19 crisis is emerging. Fiaschi and Tealdi (2022) find that the pandemic has disproportionally affected female employment in Italy. One reason is that the lockdowns have forced women with children to stay home to care for their young children, which is consistent with the largest drop in female employment in the age group between 30 and 40 years. Barth et al. (2021) report that job posting for young people with lower levels of education have declined the most since early 2020. This has affected the employment probabilities of those workers in Norway significantly. Stevenson (2020) and Winters (2021) show that job loss in the United States has been most severe for young workers with lower levels of education. Three studies have been carried out in the Netherlands. Hassink et al. (2021) study the regional impact of COVID-19 by using the severity of the number of infections as a predictor for labour-market outcomes. They find an overall negative effect on employment, which is unrelated to the number of infections in a labour-market region. Von Gaudecker et al. (2020) study the evolution of hours of work in the period February to June 2020. They report a drop in hours of work and substitution between work from home and at the workplace. After the lockdown measures are eased labour supply recovers quickly for all workers. Finally, Balgova et al. (2021) study search behaviour. They find—using survey data—that job search during the pandemic differs from previous downturns. The unemployed search less than what is normally observed during a recession, while the employed search more. Expectations about the duration of the pandemic seem to play a key role in explaining job search effort for the unemployed. Our results add to this literature by studying short-term employment probabilities of labour-market entrants in the Netherlands.

The paper is organised as follows. Section 2 documents the measures taken during the COVID-19 crisis in the Netherlands. Section 3 describes the administrative data employed in this research. Section 4 shows the empirical results. Section 5 concludes.

## COVID-19 Crisis in the Netherlands

The spread of the coronavirus (SARS-CoV-2) that causes the disease COVID-19 has radically changed the world from January 2020 onwards.^2^ In January 2020, the House of Representatives in the Netherlands was informed for the first time about an outbreak of the virus in Wuhan (China). In February 2020, the coronavirus spreads quickly in Europe and the first two Dutch infections were officially diagnosed. This health crisis has led the government to take strong measures to prevent the virus from spreading fast and to compensate employers for loss of turnover.

At the end of February 2020, the number of infections in Europe (especially in Italy and Spain) was rising rapidly and the Dutch cabinet was taking a number of measures. Corona patients were advised to stay at home and at the beginning of March a number of schools and universities were closed and air traffic became limited. In the letter of 12 March 2020, the ministers of Economic Affairs and Climate, Finance and Social Affairs and Employment together announced a number of measures to support the supply side of the economy which was hurt due to supply-chain disruptions and shortage of liquidity among several firms. These measures compensated for a drop in demand and deployed automatic stabilisers. At that point, the support included already existing possibilities for companies to apply for short-time work schemes, a new and expanded guarantee scheme for banks and non-bank lenders, using public funds absorbing extra expenses (unemployment and medical costs) and lower income without making cutbacks, the possibility for companies and entrepreneurs to defer tax payment and the monitoring of financial institutions.^3^ From that point in time onwards, there was a partial lockdown of the economy in place, which meant that for example restaurants, schools, childcare and nursing homes were closed. An emergency package of measures was put together to protect jobs and incomes quickly alongside increased hospital capacity, the purchase of medical devices and expanded test capacity.

Chronologically, our study of different cohorts starts with the preparation of the emergency package in which the Temporary Emergency Bridging Measure for the Preservation of Employment (NOW in Dutch) is the first instrument that was implemented. The goal of the package was to help employers pay salaries in absence of any revenue. The NOW was supplemented with the emergency counter for the Compensation of Entrepreneurs in Affected Sectors (TOGS), the Temporary Scheme for Self-Employed Entrepreneurs (TOZO) and the extension of a number of financing instruments for capital investments. Over time, a number of measures and specific regulations have been added, measures that have already been taken have been expanded (for example, TOGS has switched to the subsidy scheme for Compensation for Fixed Costs (TVL)) or specific groups of companies have been helped, for example by distinguishing between size (such as credit for small firms), activity (such as loans) and sector (such as a measure to pay for fixed costs among agricultural companies). These measures have been in place until 31 March 2022 and cover the entire period for which data are at our disposal (until June 2021).

The COVID-19 crisis is a health crisis, which sets it apart from a more ordinary economic recession or downturn. The main difference between this pandemic and more ordinary economic downturns is that the government took relatively extreme measures to prevent unemployment, that lockdowns lead to different shocks in labour demand and supply and that different groups have probably been benefitting and suffering more than during an economic downturn.

During the periods of lockdown the Dutch government took measures to prevent employers from releasing employees. Despite the severe drop in GDP (growth), these measures have prevented unemployment rates to go up dramatically. One could argue that—given the GDP-shock—employment has remained fairly stable because most of the wage costs of employers were compensated for. For labour-market entrants the situation is likely to be somewhat different. Entrants are more likely to work on temporary contracts, which are not extended when the economic outlook is negative. Especially the 2019 cohort was hit by surprise in early 2020, which has had an impact on their labour-market success. In terms of labour supply, potential entrants could be more reluctant to enter the labour market in the fall of 2020. When the short-term employment outlook is negative, entrants could opt to continue studying to postpone labour-market entrance. Also, during the lockdowns many students have suffered delays because they could not do an internship or were less effective in completing courses and final assignments. This results in fewer labour-market entrants in 2020.

Parts of the economy seem to not have suffered from the pandemic and some sectors (such as health care) have even increased labour demand during the COVID-19 crisis. For labour-market entrants these different effects by sector have heterogenous effects on both the employment probabilities and the decision to enter the labour market. Finally, this differential impact could have impacted the employment probabilities by gender because women are more likely to work in for example health care and education.

## Data and Statistics

The empirical analysis makes use of the administrative data from the Social Statistical Database (SSB) of Statistics Netherlands. These data are accessible for registered researchers through remote-access facilities at Statistics Netherlands. The SSB contains detailed individual-level information about completed levels of education, employment status, job characteristics, income sources and other personal and socioeconomic characteristics of all citizens of the Netherlands. Table 4 in the Appendix presents a list of all variables used in the empirical analyses and their definitions. We have access to data about labour-market developments at the individual level until June 2021.

### Number of Graduates

For the analysis in this research, we select graduates from full-time higher and secondary vocational education. We define graduates as those who enter the labour market after finishing their education and having obtained a diploma. Graduates are registered in education in the year of graduation and have entered the labour market by October of the same year (which means that they are not registered students anymore when the new education year starts in the fall). Table 1 presents an overview of the number of observation by level of education. Higher education graduates consist of three groups: university graduates who completed their masters’ education, university graduates who completed their bachelors’ education and graduates from higher vocational education who completed their bachelors’ education. Graduates from secondary vocational education have been divided into those who completed work-based education (BBL) and those who completed a school-based curriculum (BOL).^4^ Furthermore, they are divided into two levels each: higher levels refer to level 3 and 4, which is a more advanced level of education and lower levels refer to level 2 which is equal to a so-called starting qualification. We only select graduates who can be considered qualified labour-market entrants and exclude graduates from secondary vocational education level 1 from the sample.

**Table 1:** Number of observations by level of education, 2017–2020

Level of education	Year entering the labour market after completing education			
	2017	2018	2019	2020
*Higher education*				
University (Ma)	24,888	25,347	26,529	26,800
University (Ba)	9054	8828	8835	7850
Higher vocational education (Ba)	47,798	49,441	47,859	46,652
*Secondary vocational education*				
Work-based pathway				
Higher level	15,884	16,011	17,746	18,548
Lower level	6673	6339	6628	6776
*School-based pathway*				
Higher level	57,890	57,946	57,126	50,718
Lower level	14,139	13,621	13,540	12,130
Total number of observations in the empirical analysis	176,326	177,533	178,263	169,474
Total number of labour-market entrants	233,924	238,020	240,049	221,039
Percentage in empirical analysis	75.3	74.6	74.3	76.7

The year of entering the labour market after completing education is defined as having obtained a diploma and no longer registered in education by October

*Source*: Social statistical database, Statistics Netherlands

Table 1 also presents the four cohorts we consider in the analysis. We include those labour-market entrants who graduated as of October 2017–2020. This allows us to study cohorts who entered the labour market before (2017 and 2018) and during the COVID-19 crisis (2019 and 2020). The 2019 cohort is of special interest because these labour-market entrants have been surprised by the COVID-19 crisis and have been unable to adjust their behaviour. Graduates in the 2020 cohort are likely to have been able to adjust their behaviour to some extent. The adjustment could have been forced, due to study delay resulting from bottlenecks in finding internships or in hybrid education facilities which were less effective immediately after implementation. The adjustment could also have been voluntary in the sense that less promising labour-market prospects could lead to the decision to stay in education and start a new study to add more capital to the already acquired stock of human capital.

The number of observations in Table 1 show that the number of graduates from higher education are between 81,302 in 2020 and 83,616 in 2018. In terms of percentages, higher education graduates make up between 46.3 (2017) and 47.9 (2020) percent of the total number of labour-market entrants in our sample. Graduates from secondary vocational education are divided into two groups. The largest group of graduates comes from school-based tracks, between 62,848 in 2020 and 72,029 in 2017. It is clear from Table 1 that the number of graduates from these school-based tracks in 2020 is substantially lower than in the period 2017–2019. In percentages of all graduates in our sample it drops from about 40% in the period 2017–2019 to 37.1% in 2020. By contrast, the number of graduates from the work-based tracks is growing over the whole period, from about 22,500 in 2017 and 2018 to 25,324 in 2020.

The total number of labour-market entrants is larger than the number of observations in our sample. The final rows in Table 1 show the total inflow of new entrants and the percentage in our sample. Interesting to observe is that the total number of entrants increases from 2017 to 2019 and drops in 2020. In general, about 75% of all new entrants are in our sample. Entrants not in our sample are those who enter the labour market with a diploma from secondary vocational education level 1, dropouts from higher education and graduates from special needs education. Especially the number and share of dropouts from higher education is lower in 2020, suggesting that they remain in education.

### Employment Probabilities

**Fig. 1:**
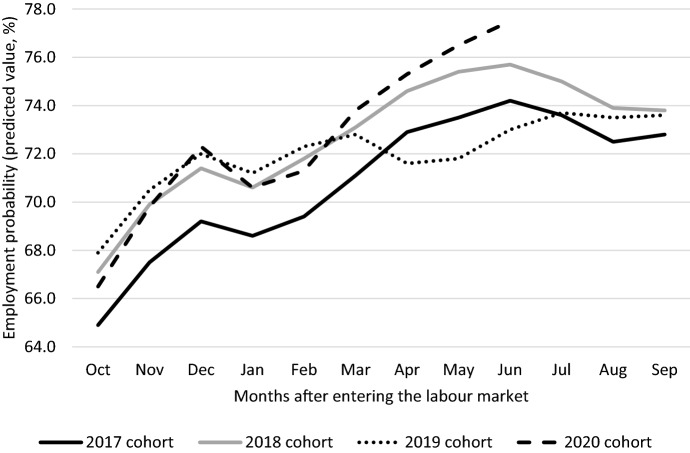
Employment probabilities of young graduates between one and twelve months after entering the labour market, 2017–2020. The year of entering the labour market after completing education is defined as having obtained a diploma and no longer registered in education by October. Employment probabilities are measured as the percentage of graduates who have obtained a job of at least three days a week. The data at our disposal are available until June 2021. T-tests reveal that relative to the 2017 cohort all employment probabilities are statistically different, with the exception of July of the 2019 cohort. Source: Social statistical database, Statistics Netherlands

Figure 1 displays the average employment probabilities by month of all graduates from the cohorts 2017–2020. Employment is defined as having a job (or multiple jobs) for at least three days (parttime work factor of 0.6) a week. For each of the four cohorts, the employment probabilities are documented in the period up to a year after graduation. In the results documented in Fig. 1, no covariates have been included. We have used logit and OLS techniques to estimate employment probabilities and both techniques lead to economic similar and statistically identical results. Since the goal of the research is to document and interpret differences between cohorts, OLS estimates are more intuitive. We therefore report OLS estimates of the employment probabilities in the remainder of the paper.

The development of employment probabilities is positive over time for each cohort, suggesting that it takes some time to find a job after entering the labour market. The 2017 and 2018 cohorts enter the labour market in a relatively positive economic environment. Economic growth is relatively high and the labour market is relatively tight in 2017 and 2018. Comparison of 2017 and 2018 suggests that the employment probabilities increase between both years, which is consistent with the economic boom in the Netherlands. The same holds for the 2019 cohort until February 2020 (see Fig. 1). Each employment probability for the 2019 cohort between October and February is higher than the employment probability of the 2018 (and 2017) cohort.

In March 2020 the first lockdown is announced and employment probabilities drop in absolute terms from April onwards and in relative terms (when comparing with the previous two cohorts) immediately after the lockdown is a fact. This drop in employment probabilities reflects the fact employers become uncertain about the future and are reluctant to extent temporary contracts. Labour-market entrants are more likely to work on a temporary contract, which means that their prospects become worse. The first lockdown runs until June 2020. During the summer, the lockdown measures become less strict and employment probabilities increase rapidly. In September 2020 employment probabilities for the 2019 cohort are comparable to the 2018 cohort one year earlier. This suggests that the labour market for young entrants has recovered relatively fast, although the employment probabilities are not yet reflecting the underlying tight labour market in the sense that they are higher than the year before.

For the 2020 cohort we have access to data from October 2020 until June 2021, that is up to nine months after entering the labour market. When these people graduated, they entered a labour market in which lockdown measures were in place. In the period between October 2020 and February 2021 there was a second lockdown in the Netherlands. Figure 1 also documents the employment probabilities of graduates who entered the labour market from October 2020 onwards. Until February 2021 their employment probabilities are lower compared to the three previous cohorts who entered the labour market in 2017, 2018 and 2019. From March onwards, employment probabilities rise and in June 2021 employment probabilities are at the highest level (77.5%) across the four cohorts. This suggests that labour demand has recovered relatively fast after the lockdown measures were (partially) cancelled and measures against the spread of the virus were eased. It seems to be the case that selection plays a role for those who enter the labour market in 2020. The statistics documented in Fig. 1 do not take this into account.

### Selection

By estimating the employment probabilities and taking into account the composition of each cohort, we attempt to control for the potential selection effects. In our analyses we include covariates with respect to gender, age, migration background, level and type of education, health status, the socio-economic status of parents (income level, social benefit use and level of education), the position of the recent graduate in the household and the province of residence.

One concern is that labour-market entrance of the 2020 cohort is selective because of the limited employment opportunities in some sectors and occupations and uncertainty about employment prospects in general. The descriptive statistics in Table 1 suggest that fewer people have entered the labour market in 2020. We test to what extent differences in covariates exist among entrants by comparing graduates who have obtained a job one month after graduating in 2019 (when people were unaware of the COVID-19 crisis) and 2020. We use employment status one month after graduating because this seems to best reflect the way entrants perceive their labour-market opportunities upon entrance. Table 5 in the Appendix documents the statistical differences by covariate.

It turns out that more women and fewer men have obtained a job immediately after graduating when comparing 2019 to 2020. Also differences are present between 2019 and the two previous cohorts. There are also differences by age, which suggest that employment opportunities for older graduates are relatively better in 2020 (worse in 2019). Again statistical differences are also present compared to the previous two cohorts. Graduates with a Dutch background are better off in 2020. Compared to 2019, their employment probabilities have increased relative to non-Dutch graduates, a difference not present between the other cohorts. Consistent with the age pattern, higher educated graduates seem to have higher employment opportunities compared to lower educated graduates in 2020. Also the family background characteristics are different. In general, graduates with more favourite background characteristics have suffered less in terms of employment opportunities. These differences are also present in comparison to the 2017 and 2018 cohorts. Finally, the province of residence leads to different employment opportunities across all cohorts.

In conclusion there seems to be selective entrance in terms of the number of graduates in 2020 (Table 1) and there are differences in background characteristics of those who have obtained a job immediately after entering the labour market. In the remainder of the analysis we include a full set of covariates to try to mitigate these potential selection effects.

## Results

In this section we present the estimation results of models in which we predict employment probabilities by including a full set of covariates.

### Adjusted Employment Probabilities

**Fig. 2:**
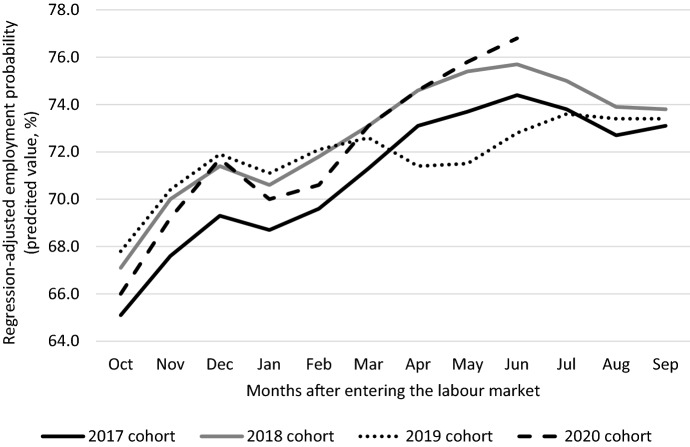
Regression-adjusted employment probabilities of young graduates between one and twelve months after entering the labour market, 2017–2020. The year of entering the labour market after completing education is defined as having obtained a diploma and no longer registered in education by October. Employment probabilities are measured as the percentage of graduates who have obtained a job of at least three days a week. The data at our disposal are available until June 2021. T-tests reveal that relative to the 2017 cohort all employment probabilities are statistically different, with the exception of July of the 2019 cohort. Source: Social statistical database, Statistics Netherlands

Figure 2 displays the regression-adjusted employment probabilities by year for all cohorts between 2017 and 2020. We estimate employment probabilities for persons in the different cohorts in each of the twelve first months after completing education. Relative to the 2017 cohort employment probabilities are higher, except when there are economic lockdowns. This reflects the underlying macroeconomic conditions of a relatively tight labour market with an average rising employment opportunities for labour-market entrants. All coefficients by month are statistically different from the 2017 cohort.

The lockdown in March 2020 has an effect on the employment probabilities of the 2019 cohort. Employment probabilities were until March 2020 comparable between the 2018 and 2019 cohort, but worsened during the lockdown. Relative to the 2018 cohort the probabilities for the 2019 cohort recovered during the Summer of 2020, and are comparable again in September. The 2020 cohort graduated during a period of economic lockdown, which has an impact until about March 2021. The curfew in January 2021 worsened employment probabilities of the 2020 cohort but probabilities recovered relatively quickly. In June employment probabilities for the 2020 cohort were higher compared to the three previous cohorts.

Figure 2 suggests a labour market that becomes tighter over time. The underlying macroeconomic situation before the pandemic in the Netherlands was one of an economic boom. The latest forecast of the CPB (2019) predicted an unemployment level below 4% in 2020 which is the lowest since the Great Recession of 2008. Based on the employment probabilities for the 2017 and 2018 cohorts we construct counterfactual probabilities for the 2019 and 2020 cohorts to measure the (temporary) loss of employment among labour-market entrants. We proceed in the following way.

**Fig. 3:**
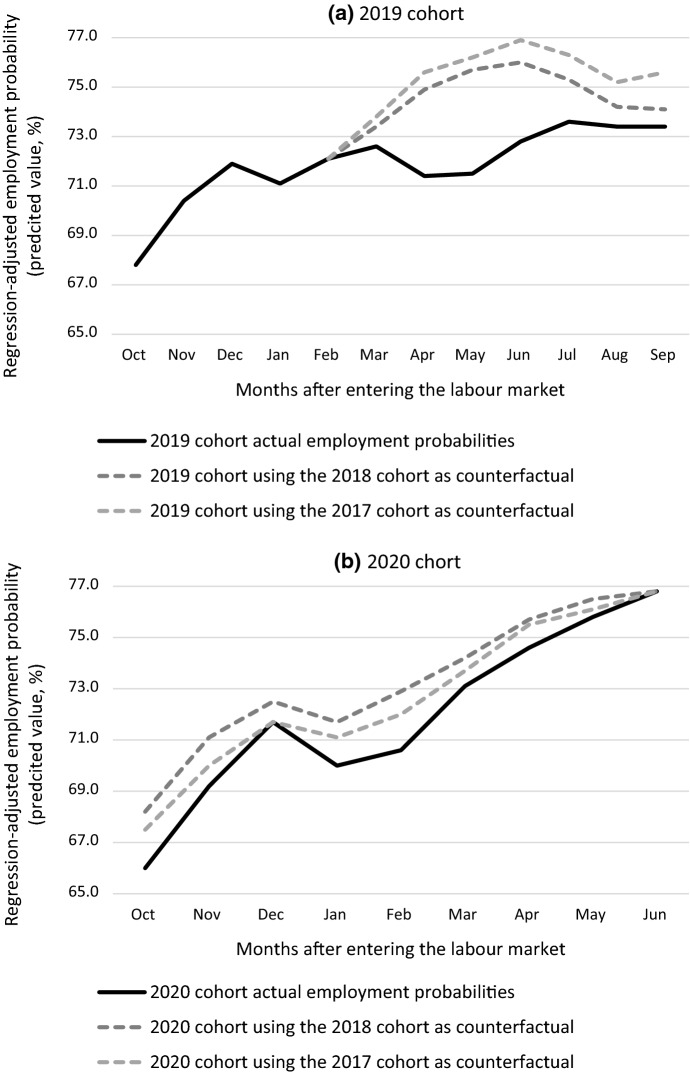
Counterfactual employment probabilities estimated based on previous cohorts. Source: Social statistical database, Statistics Netherlands

For the 2019 cohort we measure probabilities until February in a period without COVID-19. In the period March-September the 2019 cohort’s employment probabilities were substantially influenced by the economic lockdowns. We use the trends in employment probabilities from the 2017 and 2018 cohorts to predict counterfactual employment probabilities. Figure 3a shows the results. The figure first shows the actual employment probabilities, as in Fig. 2, for the 2019 cohort. The two dotted lines are the counterfactual employment probabilities based on the 2017 and 2018 trends. In June, nine months after entering the labour market the difference between the actual and counterfactual employment probabilities peeks. The difference lies between 3.2 and 4.1% points, which is the equivalent of between 5700 and 7300 jobs. In September (when entrants are one year on the labour market) the difference is reduced to between 1250 and 3900 jobs.

Figure 3b reports a similar analysis for the 2020 cohort. This cohort entered the labour market during a period of lockdown which was complemented by a curfew in January 2021. By June the strict measures were abandoned and the labour market recovered. When we carry out the same counterfactual analyses but take June as the point of departure and move back in time, the pattern in Fig. 3b results. The differences peak in October (between 1.5 and 2.2% points) and in February when the curfew was in place (between 1.4 and 2.3% points). At the point of labour market entry in October this suggests a reduction in employment between 2500 and 3700 jobs and in the midst of the curfew it boils down to between 2400 and 3900 jobs.

### Employment Probabilities by Level of Education

Table 2 reports employment probabilities by level of education in which we control for background characteristics (see Table 4). The table consists of three panels in which employment probabilities are displayed after six, nine and twelve months on the labour market. The first column with results shows the employment probabilities for graduates in the 2017 cohort. The next three columns display the differences between the employment probabilities of the 2017 cohort relative to the other cohorts.

**Table 2:** Employment probabilities by level of education

		Probability	Change in probability (percentage points) relative to the 2017 cohort		
		2017	2018	2019	2020
*After six months (March)*					
Higher education		73.8	1.86 (0.002)***	1.92 (0.002)***	4.75 (0.002)***
Secondary vocational education	Work-based higher level	89.4	0.46 (0.003)	0.36 (0.003)	− 0.55 (0.003)*
	School-based higher level	67.1	2.26 (0.003)***	1.21 (0.003)***	− 0.43 (0.003)
	Work-based lower level	79.1	− 0.08 (0.007)	− 0.16 (0.006)	− 2.52 (0.007)***
	School-based lower level	49.1	1.56 (0.006)***	0.10 (0.006)	− 2.78 (0.006)***
*After nine months (June)*					
Higher education		77.1	1.47 (0.002)***	0.04 (0.002)	4.19 (0.002)***
Secondary vocational education	Work-based higher level	89.8	0.37 (0.003)	− 0.41 (0.003)	0.24 (0.003)
	School-based higher level	70.6	1.64 (0.003)***	− 3.32 (0.003)***	1.49 (0.003)***
	Work-based lower level	80.9	− 1.28 (0.007)*	− 2.55 (0.007)***	− 1.28 (0.007)*
	School-based lower level	53.1	0.80 (0.006)	− 5.41 (0.006)***	− 0.60 (0.006)
*After twelve months (September)*					
Higher education		76.9	0.81 (0.002)***	1.25 (0.002)***	NA
Secondary vocational education	Work-based higher level	88.4	0.34 (0.003)	0.29 (0.003)	NA
	School-based higher level	67.5	0.86 (0.003)***	− 0.06 (0.003)	NA
	Work-based lower level	78.9	− 0.40 (0.007)	− 0.87 (0.007)	NA
	School-based lower level	52.1	0.76 (0.006)	− 2.24 (0.006)***	NA

The year of entering the labour market after completing education is defined as having obtained a diploma and no longer registered in education by October. Employment probabilities are measured as the percentage of graduates who have obtained a job of at least three days a week. The data at our disposal are available until June 2021

Source: Social statistical database, Statistics Netherlands

Statistical differences are defined as follows: ***1%, **5%, *10% level

Employment probabilities for higher educated graduates rise relative to the 2017 cohort of graduates. If we measure employment probabilities in March, than these probabilities rise over time. The same goes for September. In June there is a dip for the 2019 cohort, which is in the midst of the first lockdown in the Netherlands in 2020. At that point employment probabilities for the 2019 cohort are equal to employment probabilities for the 2017 cohort.

Graduates with a work-based pathway in secondary vocational education (both at the higher and lower level) seem to have relatively stable employment probabilities over time. Especially for graduates at the higher level of the work-based pathway there are no statistical differences across cohorts. Their employment probabilities are very high (around 89%) in the first year on the market and remain stable. For graduates at the lower level of the work-based pathway there is a fall in employment probabilities for the 2019 cohort in June (2.6% points) and for the 2020 cohort in March (2.6% points), part of which is still present in June. These are periods of economic lockdowns in the calendar years 2020 and 2021.

Graduates with a school-based pathway in secondary vocational education seem to be more vulnerable in terms of employment probabilities, especially at the lower level. On average they have the lowest employment probabilities as a group, and seem to suffer the most from the economic lockdowns. For graduates at the lower level of the school-based pathway we observe a substantial drop in employment probabilities of 5.4% points in June for the 2019 cohort part of which is still present in September (2.2% points). Also the drop in employment probabilities during the lockdown in March 2021 is most severe for this group (2020 cohort) of graduates.

### Employment Probabilities by Gender

Gender patterns diverge during the pandemic (Table 3). We have estimated models in which we include cohort dummies and interact those dummies with gender for each level of education.

**Table 3:** Differences in employment probabilities by cohort by gender (percentage points)

		Overall	Level of education				
			Higher education	Secondary vocational education			
				Work-based higher level	Work-based lower level	School-based higher level	School-based lower level
*2020 vs. 2017 cohort*							
After six months	Male	− 0.04	2.58	− 1.50	− 2.06	− 2.29	− 3.76
	Female	3.64	6.47	0.93	0.92	− 3.22	− 1.55
After nine months	Male	0.55	2.59	− 0.80	− 0.80	− 1.69	− 2.28
	Female	4.21	5.46	1.83	3.41	0.10	1.45
Relative difference (f-m)	Six months	3.68	3.89	2.43	2.98	− 0.93	2.21
	Nine months	3.66	2.87	2.63	4.21	1.79	3.73
*2019 vs. 2017 cohort*							
After six months	Male	0.36	1.36	− 0.53	− 0.13	− 0.78	− 1.58
	Female	2.36	2.34	1.78	2.33	1.92	2.19
After nine months	Male	− 2.29	− 0.35	− 1.15	− 4.29	− 2.81	− 6.84
	Female	− 0.82	0.34	0.76	− 2.53	− 1.63	− 3.62
After twelve months	Male	− 0.62	0.28	− 0.02	− 1.65	− 0.92	− 3.00
	Female	1.49	2.00	0.76	1.27	− 0.69	− 1.29
Relative difference (f-m)	Six months	2.00	0.98	2.31	2.46	2.70	3.77
	Nine months	1.47	0.69	1.91	1.76	1.18	3.22
	Twelve months	2.11	1.72	0.78	2.92	0.23	1.71

The year of entering the labour market after completing education is defined as having obtained a diploma and no longer registered in education by October. Employment probabilities are measured as the percentage of graduates who have obtained a job of at least three days a week. The data at our disposal are available until June 2021. Relative differences are the differences between female and male entrants after six, nine and twelve months

Source: Social statistical database, Statistics Netherlands

Between the 2017 and 2020 cohorts the average employment probability of female labour-market entrants six months after graduation grows from 70.1 to 73.7%. After nine months the growth is even 4.2% points to a level of 77.7% for the 2020 cohort. The average male figures in the same period are -0.1 and 0.6% points to levels of 72.9 and 76.3%, below the employment probabilities of women. In relative terms female labour-market entrants improve their employment probability by 3.7% points. Similar patterns emerge when we consider differences between the 2017 and 2020 cohorts in the lower panel of Table 3.

In the following columns in Table 3 we measure these differences by level of education. Focussing on the relative differences suggests that between the 2017 and 2020 cohorts the differences are rather similar with the exception of the lower level work-based graduates in secondary vocational education. It seems to be the case that the absolute employment opportunities have become less promising for those workers, both female and male. At the higher education level the numbers suggest a large absolute jump in employment probabilities of women (between 5.5 and 6.5% points) in this period.

The patterns between the 2017 and 2019 cohorts confirm the overall pattern and we are able to also analyse the differences after twelve months. The relative differences suggest that employment probabilities of women are higher after six months, after which convergence takes place. Nevertheless, differences in favour of women remain visible.

Overall, during the first year on the labour market, our estimated coefficients suggest that male graduates have seen their employment probabilities fall in relative terms during the pandemic—see also Table 5. Male labour-market entrants with lower levels of education have also experienced absolute declines in employment probabilities.

### Sectoral Differences

**Fig. 4:**
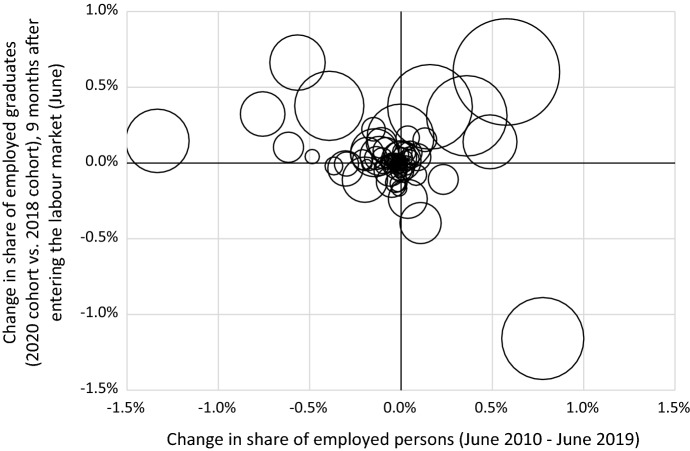
Change in share of employed graduates (2020 cohort vs. 2018 cohort) relative to the change in the share of jobs by sector of employment (June 2010–June 2019). Source: Social statistical database, Statistics Netherlands

Figure 4 plots the change in the share of employment over the last decade (2010–2019) against the change in the share of employed graduates in the period 2018–2020. The dots are scaled relative to the size of the sector and sectors are 86 two-digit sectors according to the Standard Industrial Classification (SBI 2008 in the Netherlands). The figure shows that in several sectors the share of employed graduates during the pandemic deviates from the long-term trend in the share of jobs by sector. If employment probabilities would be the same, all sectors would be on a line with similar X and Y coordinates. This suggests that the pandemic has a short-term impact on the distribution of the employment of young workers across sectors. It most likely reflects falls in labour demand induced by sector-specific lockdowns and increases in labour demand in sectors trying to mitigate the impact of the COVID-19 crisis.

For instance, the staffing industry (including temporary employment agencies, employment intermediaries and payrollers), the food and beverage industry, the hospitality industry, retail trade and healthcare are among the sectors that have seen the largest increase in the share of jobs in the decade before the pandemic. During the pandemic, the share of employed graduates only increased in healthcare and retail trade (mainly in the supermarket industry), but substantially declined in the staffing industry, the food and beverage industry and the hospitality industry. Additionally, the construction sector, the government sector, education and postal and courier services are among the sectors that have seen the largest decrease in the share of jobs in the decade prior to the pandemic. The share of employed graduates increased substantially in almost all of these sectors during the pandemic. Finally, the aviation industry and the travel industry have a rather stable share of jobs before the pandemic, but have seen a decline in the share of employed graduates during the pandemic.

This difference in employment during the COVID-19 crisis compared to the long-term trend in employment suggests that young workers have been forced to work in other sectors and probably other occupations than they would have been otherwise. It could lead to a mismatch of demand and supply in the short run, which could hamper the accumulation of human capital on the job. Given the rapid recovery of the labour market after lockdowns were eased, this seems to be particularly a short-run mismatch.

## Conclusion

This research documents employment probabilities of cohorts of graduates in the Netherlands who have entered the labour market just before and during the COVID-19 crisis and compares this to the employment probabilities of cohorts of graduates before the COVID-19 crisis. The paper focuses on the short-term (up to one year after graduation) employment probabilities of all graduates who have entered the labour market after obtaining a diploma.

The estimation results suggest that employment probabilities have fallen during the lockdowns in the Netherlands, especially for relatively lower educated graduates. The size of the employment loss seems to be relatively low when we run a number of counterfactual analyses. After measures are eased employment recovers rapidly, which reveals the underlying tight labour market in the Netherlands. Overall, the short-term employment effects of the COVID-19 crisis seem to be relatively mild compared to economic crisis when employment levels drop substantially. This could be due to the relatively extreme measures to prevent unemployment, which are often not in place during an economic crisis. Nevertheless, there are several differences in the effect of lockdowns on employment probabilities between level of education and gender. These differences could relate to sectoral and occupational differences within these groups, and thus how much labour demand is affected and the possibilities of teleworking.

Although this first and preliminary set of estimation results suggests that the effect of the COVID-19 crisis on the labour-market opportunities of the two cohorts most affected are mild, there could be long-run effects due to fewer investments in human capital or labour-market underutilisation. This could result from less investments in school during the pandemic, fewer investments made by employers in on-the-job training due to limited opportunities or underemployment or inactivity (while still being employed).

## Appendix

See Appendix Tables 4 and 5.

**Table 4:** Construction of variables

Variable	Description
Gender	All observations in the data have an indicator for gender. In the data at our disposal there are two options: male or female
Age	The age in years when graduating of a person in the database
Migration background	Distinction between graduates with a native, western and non-western background. We further distinguish between non-western migrants with a Moroccan, Turkish, Antillean and Surinamese background
	The classification of the population with a foreign background is defined by Statistics Netherlands (CBS, 2001). Classification as western or non-western is done according to country of birth. If children are born in the Netherlands, the classification is based on the mother’s country of birth and if she is also born in the Netherlands, the background is determined by the father’s country of birth. The category western includes most countries in Europe, North America and Oceania. The category non-western includes most countries in Africa, Asia and Latin-America. Individuals with a Japanese and Indonesian background are classified as western on the basis of their social and economic position in Dutch society. Individuals with a Turkish background are classified as non-western
Field of study	Variable indicating the field of study. We have information about 54 different fields of study. We merge fields of study with a low number of graduates, separately for males and females. We distinguish between 36 different fields of study for males and between 31 different fields of study for females
Level of education	Variable indicating the level and type of secondary vocational education (MBO). Level 2 is considered the lower level and levels 3 and 4 the higher level. There are two types: a school-based and work-based pathway. Higher secondary vocational education (HBO) and university education are defined as workers who have obtained diplomas from HBO and university
Health status	Variable indicating whether a person has health care costs in a given year. This could be either costs for mental health care (GGZ) or physical health care (Zorgverzekeringswet, Zvw) or both
Diploma in current year	Dummy variable indicating whether last education is finished and a diploma is received in the same year as the cohort. This is not the case when someone already received a diploma at a lower or similar level in a previous year but did not (yet) finish his/her last education at a similar/higher level
Position in household	Categorial variable indicating the home situation upon graduation. The home situation is measured three months prior to the official graduation date
Parental income decile	The dataset allows us to link children to their parents. We use information on household income as collected by the Dutch tax authorities to determine the parental income decile (compared to all households in the Netherlands). We take the median income decile in the four years prior to graduation and use information of both parents if available. We average income if parents have separate households. Parental income refers to the standardized disposable income (the net amount a household is able to spend on an annual basis), adjusted for differences in household size and composition. Parental income is not available for graduates without parent(s) required to pay income tax in the Netherlands
Primary source of income parents	Combining information from different income sources, Statistics Netherlands determines the primary income source of households in the Netherlands. We determine the primary income source of parents by looking at the most common primary income source in the four years prior to graduation. We use information of both parents if available. We randomly select one parents’ primary income source if both parents live in separate households. Primary income source is not available for graduates without parent(s) required to pay income tax in the Netherlands
Parental education	Categorial variable indicating the highest attained education of parents. Statistics Netherlands combines information from the registration of graduates (Dutch: DUO), the Employee Insurance Agency (Dutch: UWV) and the annual Labour Force Survey (Dutch: EBB) to determine the educational attainment levels of the population in the Netherlands, as educational attainment levels are not available in administrative data for most older people. The parent with the highest educational attainment determines the value of parental education. Classification is based on the Dutch classification of education levels (SOI 2016), which uses International Standard Classification of Education (ISCED) as a starting point. For the most recent cohorts (2014–2016), parental education levels are available for 70% of graduates
Province of residence	Province of residence upon graduation, measured three months prior to official graduation date

**Table 5:** Difference in employment and unemployment status one month after entering the labour market, 2017–2020

Employed after one month (yes = 1; no = 0)	2017		2018		2019		2020	
	1	0	1	0	1	0	1	0
*Gender*	*	*	*	*			*	*
Male	49.6	44.3	49.1	44.6	49.8	46.1	48.8	47.8
Female	50.5	55.7	50.9	55.4	50.2	53.9	51.2	52.2
*Age*	*	*	*	*			*	*
16	0.0	0.0	0.0	0.0	0.0	0.0	0.0	0.0
17	0.0	0.0	0.0	0.0	0.0	0.0	0.0	0.0
18	1.9	2.3	1.8	2.3	1.9	2.6	1.9	2.9
19	7.0	8.4	7.3	8.5	7.9	9.1	7.6	9.4
20	10.7	11.2	10.3	10.8	10.8	11.1	10.4	11.4
21	12.7	13.6	12.7	13.6	12.4	13.5	12.4	12.9
22	12.9	13.5	12.7	13.5	12.5	13.2	12.1	12.8
23	12.9	13.5	13.0	12.8	12.3	12.5	12.6	12.3
24	12.5	11.8	12.6	12.1	12.4	12.0	12.5	11.4
25	10.6	9.4	10.8	9.8	10.8	9.5	10.9	9.8
26	8.0	6.6	7.9	6.7	7.8	6.8	8.1	6.9
27	5.2	4.6	5.3	4.6	5.3	4.7	5.4	4.8
28	3.4	3.1	3.5	3.2	3.6	3.1	3.8	3.3
29	2.2	2.0	2.2	2.1	2.4	2.1	2.5	2.2
*Migration background*	*						*	*
Dutch	82.9	73.1	82.6	72.6	82.3	72.6	83.0	72.5
Morocco	2.2	3.7	2.2	3.9	2.2	4.0	2.1	3.8
Turkey	2.5	4.2	2.5	4.3	2.7	4.3	2.5	4.1
Surinam	2.1	3.4	2.1	3.5	2.1	3.4	1.9	3.3
Dutch Antilles & Aruba	1.1	2.0	1.2	1.9	1.3	1.8	1.1	1.7
Non-western	3.9	6.5	4.1	6.6	4.2	6.8	4.2	7.3
Western	5.3	7.2	5.3	7.2	5.2	7.1	5.2	7.2
*Level of education*	*	*	*				*	
High	46.4	46.8	47.1	47.6	46.4	47.4	48.5	46.9
Work-based higher level	12.3	2.8	12.1	2.7	13.2	3.0	14.7	3.5
School-based higher level	31.0	35.8	31.2	35.2	30.5	35.0	27.7	34.3
Work-based lower level	4.7	2.2	4.3	2.2	4.4	2.4	4.5	3.0
School-based lower level	5.6	12.4	5.4	12.2	5.4	12.2	4.6	12.3
*Health problems*	*	*	*	*			*	*
No health problems	79.4	76.9	79.5	76.7	79.3	77.0	79.5	77.3
Physical health problems	15.4	15.4	15.0	15.3	14.8	14.8	14.5	14.2
Mental health problems	3.7	5.0	3.8	5.3	4.2	5.6	4.3	5.7
Both	1.5	2.6	1.7	2.7	1.7	2.6	1.7	2.8
*Diploma in current year*	*	*					*	*
No	13.3	20.1	14.5	21.1	14.7	21.5	12.7	19.5
Yes	86.7	79.9	85.5	78.9	85.3	78.5	87.3	80.5
*Parental education*	*	*	*	*			*	*
Low	13.3	15.1	13.2	14.9	13.0	14.4	12.1	14.3
Middle	31.1	28.9	31.4	28.6	31.6	28.8	31.2	29.0
High	26.9	30.6	27.7	32.2	28.8	33.6	30.2	33.6
Unknown	28.7	25.4	27.8	24.3	26.6	23.2	26.5	23.2
*Social benefits parents*	*	*	*	*			*	*
No	91.6	87.5	91.8	87.8	92.3	88.5	93.2	89.0
Yes	8.4	12.5	8.2	12.2	7.8	11.5	6.8	11.0
*Parental income*	*	*	*	*			*	*
Decile 1	1.7	3.5	1.7	3.6	1.6	3.5	1.6	3.4
Decile 2	4.1	7.5	4.2	7.3	4.0	7.0	3.6	6.7
Decile 3	4.6	6.4	4.6	6.4	4.3	6.1	4.0	6.2
Decile 4	6.6	7.7	6.5	7.9	6.5	7.8	6.1	7.9
Decile 5	9.6	9.8	9.4	9.8	9.6	9.8	9.1	9.9
Decile 6	12.0	11.2	12.0	11.1	12.2	11.2	12.0	11.7
Decile 7	14.1	12.0	14.0	11.8	13.9	11.8	14.4	12.6
Decile 8	15.3	12.4	15.6	12.1	15.7	12.8	15.8	12.7
Decile 9	16.2	13.2	16.2	13.2	16.3	13.4	16.9	12.9
Decile 10	14.6	14.6	14.6	15.4	14.8	15.0	15.5	14.4
Unknown	1.0	1.7	1.1	1.6	1.1	1.6	1.1	1.7
*Position in household*	*	*	*	*			*	*
With parents	43.6	44.0	43.9	44.0	45.0	45.5	44.7	46.0
With one other parent	9.2	13.0	9.7	13.7	10.0	14.0	9.9	14.5
In different family	0.7	1.0	0.7	0.9	0.6	0.9	0.6	0.8
Single without children	19.5	19.7	19.4	20.0	18.8	19.4	18.9	18.8
Together without children	21.8	14.1	21.4	13.5	20.6	13.0	21.2	13.1
Single parent	0.5	1.4	0.4	1.3	0.4	1.1	0.4	1.1
Together with children	2.1	2.8	2.0	2.6	2.0	2.4	2.0	2.2
Rest	2.4	3.0	2.4	3.0	2.4	2.8	2.0	2.6
Institutional household	0.2	1.1	0.2	1.0	0.2	0.9	0.2	0.9
*Province of residence*	*	*	*	*			*	*
Drenthe	2.3	2.2	2.3	2.0	2.4	2.4	2.6	2.2
Flevoland	2.2	2.4	2.2	2.6	2.2	2.4	2.2	2.7
Friesland	3.5	3.8	3.6	3.6	3.7	3.7	3.8	3.6
Gelderland	12.5	11.3	12.5	11.2	12.7	11.2	13.0	11.2
Groningen	4.3	5.3	4.4	5.2	4.4	5.4	4.3	4.8
Limburg	5.7	5.2	5.6	4.9	5.4	4.8	5.5	4.9
Noord-Brabant	15.0	12.6	14.8	12.2	15.1	12.5	15.0	12.8
Noord-Holland	16.2	18.9	16.0	19.0	15.5	18.5	15.0	18.8
Overijssel	7.4	6.0	7.5	6.2	7.5	6.2	7.6	6.2
Utrecht	8.6	9.0	8.5	8.9	8.5	9.3	8.6	9.0
Zeeland	1.9	1.4	2.0	1.4	2.0	1.4	2.0	1.4
Zuid-Holland	20.4	21.8	20.7	22.7	20.6	22.3	20.4	22.4
Number of observations	62,452	115,609	58,963	120,141	57,606	122,081	56,727	112,747
Share of graduates by cohort	35.1	64.9	32.9	67.1	32.1	67.9	33.5	66.5

Statistical differences are measured by conducting a chi-square test by variable/covariate to determine the differences with regard to 2019

Source: Social statistical database. Statistics Netherlands

*Significant difference in composition cohort for this characteristic compared to 2019 cohort

## Footnotes

1 Van Ours (2015) analyses a number of recessions and concludes that young workers are most affected. Von Wachter (2020a) summarises the literature about the long-term and short-term effects of graduating in a recession. Von Wachter (2020b) focuses on the possible effects of the COVID-19 crisis and suggests a number of policy options to mitigate the negative effects on youth employment.

2 COVID-19 is caused by the coronavirus SARS-CoV-2. The disease leads to respiratory problems (breathing problems) and fever. The virus is spread by droplets released when coughing, sneezing or talking. Infection occurs because these droplets end up in the mouth, nose or eyes.

3 A number of measures were also taken in a European context. The European Council mandated the Commission to take corona measures and coordinate the approach in the EU countries. 60 billion euros of unused cohesion policy funds were mobilized in March 2020 to fight the pandemic.

4 See e.g., Bisschop et al. (2020) for a more elaborate description of these groups from secondary vocational education.
